# Role of Usual Healthcare Combined with Telemedicine in the Management of High-Risk Pregnancy in Hangzhou, China

**DOI:** 10.1155/2019/3815857

**Published:** 2019-05-06

**Authors:** Xu-Hong Zhu, Jing Tao, Li-Yuan Jiang, Zhi-Feng Zhang

**Affiliations:** ^1^Department of Obstetrics and Gynecology, Hangzhou Women′s Hospital (Hangzhou Maternity and Child Health Care Hospital), No. 369, Kun Peng Road, Hangzhou, Zhejiang 310003, China; ^2^Department of Obstetrics and Gynecology, Nanjing Medical University, No. 101, Ron Mian Road, Nanjing, Jiangshu 211166, China

## Abstract

**Background:**

Maternal health is an important part of basic public health services in China's medical reform. Effective management is an important guarantee of maternal health. Telemedicine has been widely used in maternal health management.

**Objective:**

This study explores the role of usual healthcare combined with telemedicine in the management of high-risk pregnancy.

**Methods:**

The study was a retrospective. Data were obtained from Hangzhou Maternity Hospital between October 2012 and September 2016, including 93465 pregnant women who were in usual high-risk pregnancy management (usual group) and 134884 pregnant women who were in telemedicine combined with usual high-risk pregnancy management (telemedicine group). The differences in high-risk scores and pregnancy outcomes between the usual and the telemedicine groups were compared.

**Results:**

The high-risk factors were analyzed, and the results showed that the first fixed high-risk factor was scar uterus and the first dynamic high-risk factor was hepatitis B. Comparing the data of two groups, the number of prenatal visits increased significantly in the telemedicine group (*p* value <0.05). Although the critical proportion of high-risk women was 2.13% in the usual group and 5.88% in the telemedicine group, respectively (*p* value <0.01), maternal mortality decreased in the telemedicine group (*p* value <0.05).

**Conclusion:**

The combination of telemedicine and usual healthcare can urge the pregnant women to carry out antenatal visits on time, which is one of the important factors to improve the outcome of high-risk pregnancy.

## 1. Background

In recent years, with the continuous attention of Chinese government to women's health, the policy and legal system covering national macrohealth policies and women's health protection has been improved. Maternal mortality is one of the important indicators to measure the economic, cultural, and maternal and child health management of a region [1, 2]. Maternal health and infant safety are the main areas of healthcare. It is reported that more than 80% of maternal deaths can be avoided by strengthening maternal healthcare [3, 4].

Hangzhou is the capital of Zhejiang Province and works as the center of politics, economy, science, education, and culture of the province. Hangzhou has a permanent population of 9188,000 in 2016. There are 216 medical institutions with obstetrics, of which 198 are the primary medical institutions and 8 maternity hospitals. The process of pregnancy healthcare in Hangzhou is divided into 3 phases from pregnancy to 42nd day after delivery. At the first stage, 24 weeks after the pregnancy, doctors at primary medical institutions established the mother-child health manual and implemented basic maternal healthcare for pregnant women. The second stage is from 24 weeks of pregnancy to childbirth, during which pregnant women are examined and delivery in the maternity hospitals. The third stage, from delivery to 42 days postpartum, women go back home and enjoy the free services provided by the doctor of primary medical institutions [5, 6].

Since September 2015, couples in China are allowed to have two children. China's decision to implement this revised policy was stimulated by concerns related to the country's shrinking population and ageing workforce. With the enactment of China's new birth policy, high-risk pregnancies have increased dramatically [7]. For example, there has been an increase in the number of older pregnancies (older than 35 years) and scarred-uterine pregnancies.

Strong evidence demonstrates that early monitoring and intervention are the effective measures to improve the maternal health and reduce maternal and perinatal mortality. In recent years, several studies have addressed the feasibility and efficacy of telemedicine strategies on the management of high-risk pregnancy [8–10]. The strategies of telemedicine application included the computerized systems for information exchange, the video conferencing, and the exchange of information via telephone or other mobile devices, short message service, or through the Internet. Telemedicine service for high-risk pregnancy is a relatively new field of research. The conclusions of the studies are not identical. Several trials have evaluated the economic impact of telemedicine compared with conventional care, and the results show that the telemedicine can reduce health-related costs and improve benefits for women diagnosed with gestational hypertensive diseases [11] and high-risk pregnancy [12, 13]. In addition, internet-based remote monitoring for managing gestational diabetes mellitus is also a viable approach for healthcare delivery and enhances patients' health-related quality of life [14]. Finally, elevated feelings of maternal satisfaction were obtained when telemedicine was used in obstetrical care [15].

With the increasing demand for healthcare in pregnancy, the usual high-risk pregnancy healthcare has been unable to meet the needs. Challenges in the care of high-risk pregnancy require innovative management strategies. Hangzhou has established a comprehensive, cooperative, and data-sharing system for maternal health. The system is organically combined by a private cloud system and a public cloud system. In private cloud system, several obstetric electronic medical record microservice systems deployed in various hospitals exchange maternal health data in real time through message middleware in private health network. In addition to the distributed local databases in hospitals, the entire maternal health data are integrated into a central Oracle database system that is physically isolated from the Internet. At the same time, a public cloud system is designed to support applications (APP) services, which is composed of a web-based service and the mobile APPs. In order to protect security of the health data, the web-based service is deployed on a server machine which can only allow authorized access to the central maternal health database over air gapping. And, HTTPS protocol is used to implement secure data transfer and resource sharing. When APP users propose requests, the key identity information, such as the identification number and the telephone number, are transmission encrypted over SSL protocol to avoid privacy leak. Moreover, mobile phone short message service (SMS) verification and face recognition technology are used for real-name authentication.

Since the application of telemedicine in maternal health is gradually being implemented in Hangzhou city, some pregnant women were assisted by telemedicine and other pregnant women continued to use usual healthcare measures between 2012 and 2016. This study retrospectively analyzed the role of usual healthcare combined with telemedicine in high-risk pregnancy management.

## 2. Methods

### 2.1. Diagnostic Criteria for High-Risk Pregnancy

A pregnancy is considered high-risk when there are potential complications that could affect the mother, the baby, or both. High-risk pregnancies require management by a specialist to help ensure the best outcome for the mother and baby [16]. According to the Hangzhou's high-risk pregnancy management rules, the criteria for high-risk assessment include the following: (1) Fixed factors and environmental and social factors include the basic situation of pregnant women, the history of abnormal pregnancy, obstetrics and gynecology surgery, incompatible history, history of mental illness, teratogenic factors, education level, and economic condition. (2) Pregnancy comorbidity and complications include hypertension, heart disease, diabetes, nephropathy and other more than 10 kinds of pregnancy comorbidities, pregnancy-induced hypertension, abnormal fetal position, threatened abortion and other more than 10 kinds of pregnancy complications, and other diseases. According to the evaluation method of pregnant women in Hangzhou, the evaluation results are divided into four levels: no risk factor, A, B, and C. Management is stratified based on the high-risk level.

### 2.2. Management of High-Risk Pregnancy

The maternal healthcare service system has been continuously improved in China. China has established a hierarchical network of maternal health services, with each level taking a supervisory and teaching role for the level below it. This system is also called the maternal health three-level network system. A three-level network of maternal healthcare service has been put in place in urban and rural areas. The three-level maternal healthcare network system in China is based on the municipal maternity hospitals, primary medical institutions, and health service stations. According to the Hangzhou high-risk pregnancy management, if the pregnant woman is assessed as level A, the doctors in the primary medical institutions will be responsible for the management. If the pregnant women are evaluated as level B or C, the doctors in the primary medical institutions should report to the Hangzhou Maternity Hospital and the doctors in the Hangzhou Maternity Hospital are responsible for guiding the doctors in the primary medical institutions to follow up the high-risk pregnant women.

**Figure 1:**
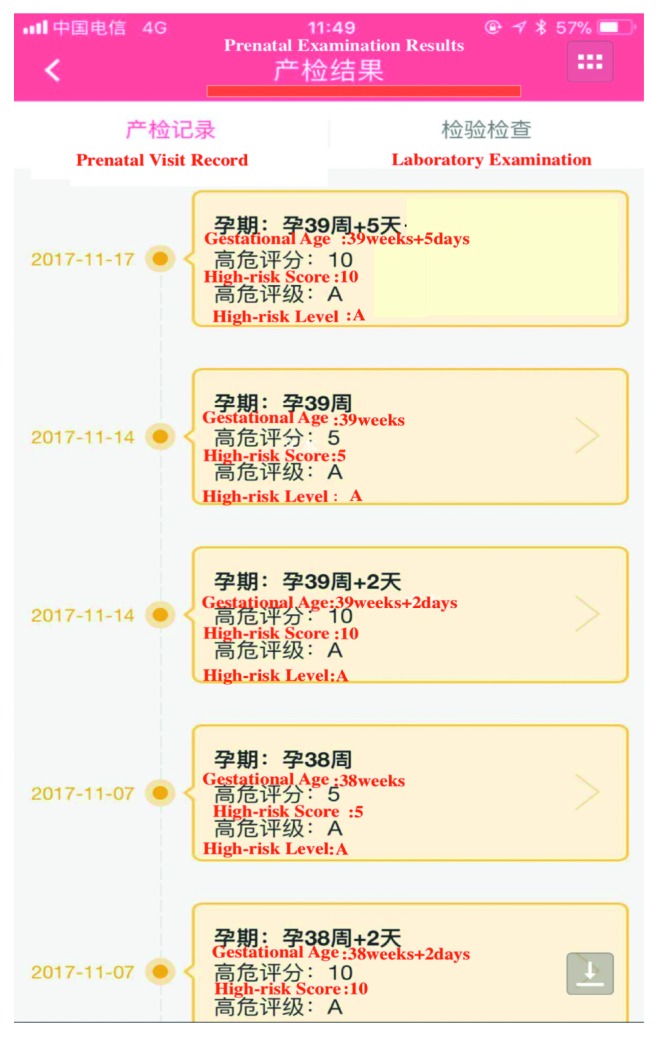
Prenatal visit record.

**Figure 2:**
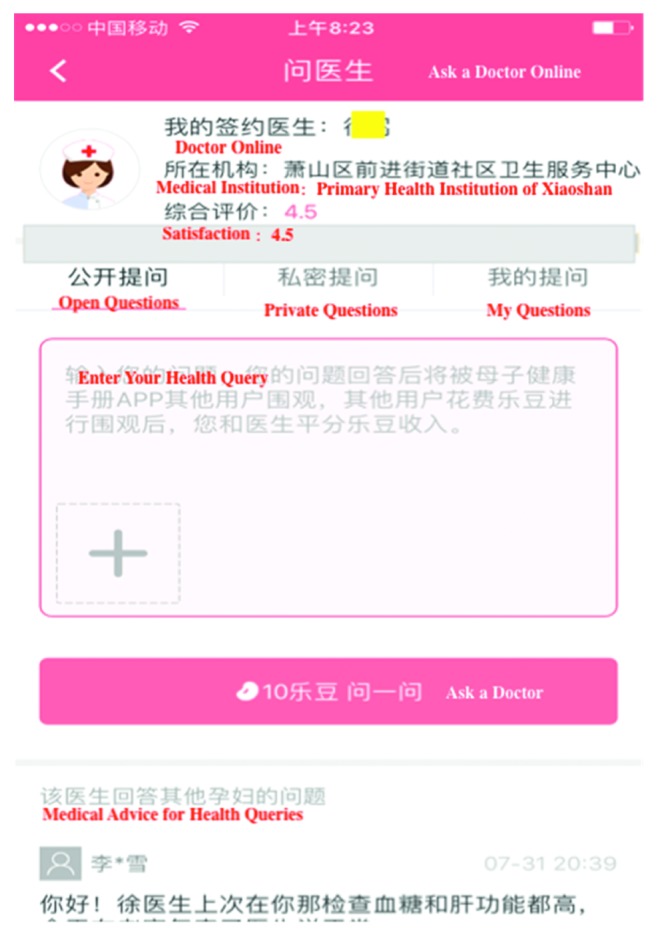
Health advice online.

**Figure 3:**
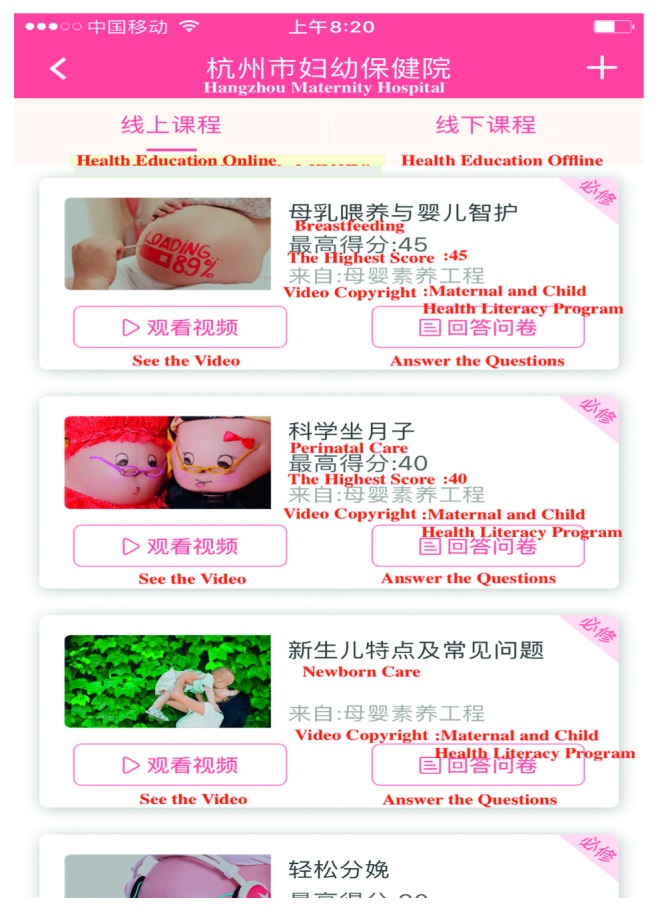
Health education online.

**Figure 4:**
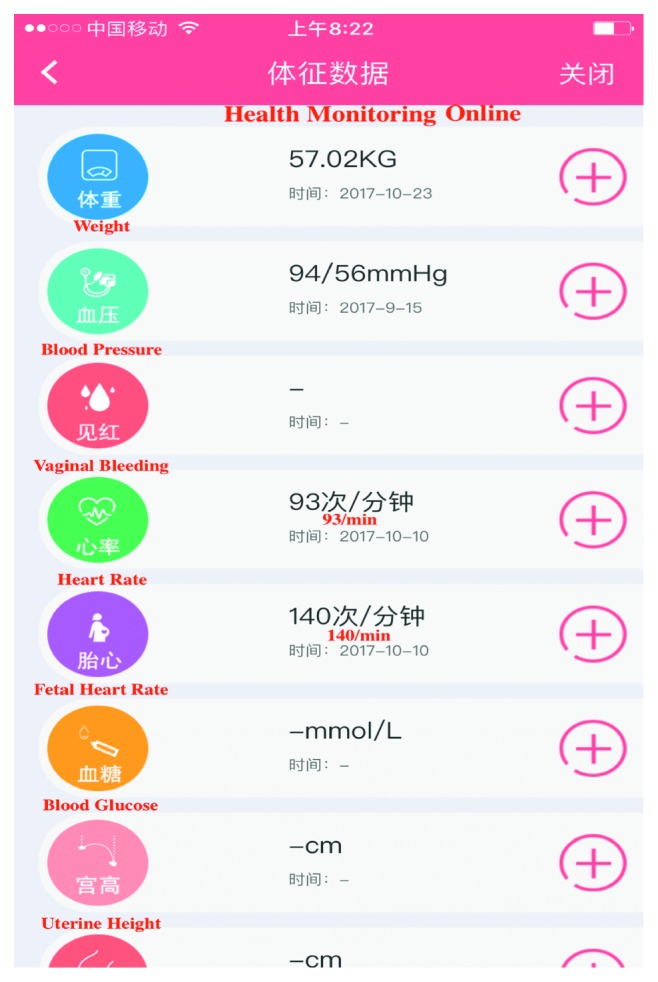
Health monitoring online.

The management of high-risk pregnancies is registered on paper, and those who have not applied telemedicine were in the usual management group. Usual healthcare for high-risk pregnancy is based on the maternal management regulations in Hangzhou [17]. The main measures include regular prenatal healthcare and periodic high-risk assessment. The telemedicine application includes computerized systems for information exchange, APP (Figures 1–4), and exchange of information via mobile devices, short message service, or through the Internet.

### 2.3. Data Source

The study was a retrospective investigation. Hangzhou Maternity Hospital is responsible for the management of high-risk pregnancy in Hangzhou city. The data of the high-risk pregnancy is obtained from the Hangzhou Maternity Hospital between October 2012 and September 2016, which is the whole population data of high-risk pregnant women in Hangzhou. The demographic characteristics are reported in Table 1.

Inclusion criteria: at least 5 antenatal visits were completed during pregnancy; high-risk score must be carried out for every prenatal visit; give birth in a midwifery hospital of Hangzhou. 93465 high-risk pregnant women were considered as the usual group. Data were collected from the high-risk maternal management register (paper record). 134884 high-risk pregnancies were considered as the telemedicine group. Data collection was carried out by the maternal information system.

During pregnancy, pregnant women at high risk are evaluated several times, and the data in this paper are the highest score in pregnancy. The study was approved by the Hospital Ethics Committee.

### 2.4. Statistical Analysis

SPSS19.0 statistical software was used for data analysis, and the counting data were tested by *χ*^2^. *p* value <0.05 was considered to be statistically significant.

## 3. Results

### 3.1. Occurrence of High-Risk Pregnant Women

In the usual group, there were 93,465 high-risk pregnant women. In the telemedicine group, there were 134,884 high-risk pregnant women (Table 2). The data of two groups of high-risk pregnant women were analyzed. The results showed in the telemedicine group, maternal ratio of ≤18 years old and ≥40 years old was significantly increased compared with the usual group (*p* value <0.05, Table 1); comparing the usual group and the telemedicine group, there was no statistical difference in the number of pregnant women and the number of antenatal visits in the A level or B level (*p* value>0.05, Table 2), but there was significant increase in the C level in the telemedicine group (*p* value <0.05, Table 2).

### 3.2. Top Five High-Risk Factors

The first high-risk factor in fixed risk factors was the scar uterus. The proportion of scar uterus was 27.88% in the usual group and 17.27% in the telemedicine group, respectively. There is a great risk in the birth of scar uterus after pregnancy, and the nonmedical indications should be strictly controlled. The first high-risk factor in dynamic risk factors was hepatitis B virus carrier (Table 3). The proportion of hepatitis B virus carrier was 9.13% in the usual group and 8.92% in the telemedicine group, respectively. Blocking the vertical transmission from mother to infant is the key to reduce the incidence of hepatitis B. It is a concern of the whole society to do a good job in blocking the vertical transmission of mother and infant.

### 3.3. Outcome of Maternal Pregnancy and Perinatal Newborns

In the usual group, the maternal mortality was 5.19 per 100,000, with a perinatal newborn mortality of 4.59‰. In the telemedicine group, the maternal mortality was 4.92 per 100,000, with a perinatal neonatal mortality of 4.36‰ (Table 4). Maternal mortality decreased in the telemedicine group (*p* value <0.05).

## 4. Discussion

Telemedicine is the use of telecommunication and information technology to provide clinical healthcare from a distance. Telemedicine application is now ubiquitous in modern society. The current telemedicine mainly consists of teleconsultation, telediagnose, tele-education, telecare, and telemedical car.

During pregnancy and in preparation for motherhood, many women seek information and try to adopt a healthy lifestyle. The recent emergence of mobile and other eHealth technologies has resulted in an increased use of these tools in health prevention- and health promotion-based intervention frameworks for varied clinical areas [18, 19].Pregnant women are increasingly using mobile APP to access information, monitor fetal development, track individual health indicators, and provide reassurance [20]. In Canada, over 300,000 women are pregnant annually, with approximately 60% exceeding evidence-based weight gain recommendations. APPs have the potential to reduce excessive gestational weight gain, offering pregnant women trustworthy guidance, ultimately improving the health outcomes of mothers and infants [21]. Depending on the information system platform, the maternal and child health handbook APP (MCH-APP) in Hangzhou ensures the contact between pregnant women and doctors. Women are monitored and managed from the date of pregnancy planning or pregnancy until 42 days after childbirth. If serious complications occur in pregnant women, real-time intervention, effective treatment, or termination of pregnancy can be performed. Meanwhile, pregnant women can check her antenatal information (Figure 1), can interact with doctors (Figure 2), and can also conduct online health education (Figure 3) and self-monitoring in APPs (Figure 4). To sum up, relying on the information platform, managers can dynamically observe the health information of all high-risk pregnant women in real-time in Hangzhou and doctors can improve their work efficiency. Throughout the pregnancy, pregnant women can check her antenatal information, can interact with doctors, and can also conduct online health education and self-monitoring in MCH-APP. The information platform realizes the real-time closed-loop management of high-risk pregnant and pregnant women. Our previous research has shown that MCH-APP may improve the self-care awareness of pregnant women [22].

Some studies have shown that telemedicine plays a role in management of high-risk pregnancy. For example, Nores et al. [23]demonstrated successful interpretation of first-trimester obstetric ultrasound, directed by a perinatologist in a satellite location. Similarly, women with potentially poor pregnancy outcomes were given diagnoses and guidance via a telemedicine consultation with a perinatologist. [24] For rural or remote settings, telemedicine offers access to specialists and subspecialists, which is an essential need for high-risk maternity and neonatal patients [25]. According to Hangzhou high-risk pregnancy management rules, all pregnant women are screened with high-risk factors. If the maternal assessment is considered to be at high-risk level A or B or C, the pregnant women will be stratified to regulate medical treatment. This management process reduces the level of maternal risk. Comparing the data of the usual group and the telemedicine group, although the critical proportion of high-risk women was 2.13% and 5.88%, respectively (*p* value <0.01), the maternal mortality was 5.19/100,000 and 4.92/100,000, respectively (*p* value <0.05). When a woman is pregnant in the telemedicine group, the pregnant woman is asked to download the MCH-APP. After the establishment of MCH-APP, the pregnant woman is asked to carry out antenatal visits on schedule in the primary medical institution. Throughout the pregnancy, the responsible doctor directs the pregnant woman to complete various prenatal visits. If a maternal abnormality is found, the responsible doctor should transfer the pregnant woman to the maternity hospital in time to ensure the safety of the pregnant woman. In the telemedicine group, high-risk pregnant women are timely assessed and given health education or medical guidance based on the use of telemedicine, which may be one of the reasons for the reduction in maternal mortality.

Evidences suggest that telemedicine can contribute in reducing the various phases of delay in obtaining help for pregnant women and improving correct management of patients [26]. High-risk pregnant women in the usual group in this study were managed by using paper records. This management approach not only leads to loss and omission of records but also requires a large number of people to do the work. In the telemedicine group, the accuracy and integrity of the data of high-risk pregnant women were improved through the information platform. When the doctor is in the process of diagnosis and treatment, the data are entered only once, but used multiple times. This not only reduces the doctor's repeated work but also ensures that the data are true and accurate. Telemedicine applications are important for planning, monitoring, and improvement of the maternal health. Telemedicine improves the management efficiency.

## 5. Conclusion

In conclusion, maternal health is an important part of basic public health services. The pregnancy stage is an important stage of the female life cycle. Hangzhou has long recognized the importance of maternal and child healthcare and has made tremendous efforts to improve the quality of such care. Telemedicine combined with usual healthcare on high-risk pregnancy is one of the important measures to ensure the health of pregnant women. Since 2017, Hangzhou's pregnant women have fully and freely enjoyed telemedicine combined with usual healthcare services.

## Figures and Tables

**Table 1:** Characteristics of the study population.

Characteristics	Usual group (*N* = 93465)	Telemedicine group (*N* = 134884)
Age (years), *N* (%)		
≤18	230 (0.24)	580 (0.43^*∗*^)
19–34	88921 (95.14)	127467 (94.50)
35–39	2880 (3.08)	3997 (2.96)
≥40	1434 (1.54)	2840 (2.10^*∗*^)
Highest education degree, *N* (%)		
Primary education	4673 (5.00)	5514 (4.09)
Secondary education	35517 (38.00)	49877 (36.99)
University degree	53275 (57.00)	79493 (58.93)
Parities, *N* (%)		
Primipara	48023 (51.38)	71875 (53.29)
Multipara	45442 (48.62)	63009 (46.71)
Gravidity, *N* (%)		
1	37753 (40.39)	55884 (41.43)
2	28057 (30.02)	50668 (37.56)
≥3	27655 (29.59)	28332 (21.00)

^*∗*^Compared with the usual group, maternal ratio of ≤18 years old and ≥40 years old was significantly increased (*p* value <0.05).

**Table 2:** Summary of high-risk pregnancy between usual and telemedicine groups.

Group	High-risk maternal	A level		B level		C level	
	*N*	*N* (%)	Prenatal visit (time)	*N* (%)	Prenatal visit (time)	*N* (%)	Prenatal visit (time)
Usual	93465	79258 (84.80)	6.52	12215 (13.07)	7.26	1992 (2.13)	8.04
Telemedicine	134884	101136 (74.98)	6.95	25816 (19.14)	8.35	7932 (5.88^*∗*^)	10.37^*∗*^

^*∗*^Compared with the usual group, pregnant women who were assessed as the C level were significantly increased (*p* value <0.05). The number of pregnant women who performed antenatal visits increased significantly compared with the usual group (*p* value <0.05).

**Table 3:** Top five high-risk factors of pregnancy.

High-risk factors		Usual group *N* = 93465 (%)	Telemedicine group *N* = 134884 (%)
Fixed risk factors	Scar uterus	26062 (27.88)	23302 (17.27)
	Miscarriage (natural, artificial) ≥2 times	10425 (11.15)	15186 (11.26)
	BMI ≤ 18	4601 (4.92)	920 (0.68)
	Age ≥ 35 years old	4314 (4.62)	6837 (5.07)
	BMI ≥ 25	3343 (3.58)	6558 (4.86)

Dynamic risk factors	Hepatitis B virus carrier	8537 (9.13)	12031 (8.92)
	Diabetes (including impaired glucose tolerance) is not required medication	2786 (2.98)	4326 (3.20)
	Normal ＜ ALT ＜ 100 IU	2355 (2.51)	4186 (3.10)
	Hypothyroidism is required medication	1977 (2.12)	5093 (3.78)
	Anemia (Hb 80–99 g/L)	1430 (1.52)	4163 (3.08)

BMI: body mass index. ALT: glutamic-pyruvic transamin.

**Table 4:** Outcome of maternal pregnancy and perinatal neonatal mortality.

	Maternal mortality (/100,000)	Perinatal neonatal mortality (‰)
Usual group	5.19	4.92
Telemedicine group	4.19^*∗*^	4.36

^*∗*^The maternal mortality declined in the telemedicine group (*p* value <0.05).

## Data Availability

The data used to support the findings of this study are included within the article. Data used to support the results of this study were provided by Hangzhou Maternity Hospital and approved by the Hospital Ethics Committee.
